# Visual cues of soft-tissue behaviour in minimal-invasive and robotic surgery

**DOI:** 10.1007/s11701-024-02150-y

**Published:** 2024-11-07

**Authors:** Robin Julia Trute, Afshin Alijani, Mustafa Suphi Erden

**Affiliations:** 1 School of Engineering and Physical Sciences, Heriot-Watt University, Edinburgh, UK; 2 Edinburgh Centre for Robotics, Edinburgh, UK; 3 Ninewells Hospital, University of Dundee, Dundee, UK

**Keywords:** Visual cues in surgery, Visual feedback in robotic surgery, Visual cues in surgical education

## Abstract

Minimal-invasive surgery (MIS) and robotic surgery (RS) offer multiple advantages over open surgery (Vajsbaher et al. in Cogn Syst Res 64:08, 2020). However, the lack of haptic feedback is still a limitation. Surgeons learn to adapt to this lack of haptic feedback using visual cues to make judgements about tissue deformation. Experienced robotic surgeons use the visual interpretation of tissue as a surrogate for tactile feedback. The aim of this review is to identify the visual cues that are consciously or unconsciously used by expert surgeons to manipulate soft tissue safely during Minimally Invasive Surgery (MIS) and Robotic Surgery (RS). We have conducted a comprehensive literature review with papers on visual cue identification and their application in education, as well as skill assessment and surgeon performance measurement with respect to visual feedback. To visualise our results, we provide an overview of the state-of-the-art in the form of a matrix across identified research features, where papers are clustered and grouped in a comparative way. The clustering of the papers showed explicitly that state-of-the-art research does not in particular study the direct effects of visual cues in relation to the manipulation of the tissue and training for that purpose, but is more concentrated on tissue identification. We identified a gap in the literature about the use of visual cues for educational design solutions, that aid the training of soft-tissue manipulation in MIS and in RS. There appears to be a need RS education to make visual cue identification more accessible and set it in the context of manipulation tasks.

## Introduction

Minimal-invasive surgery (MIS) and robotic surgery (RS) offer multiple advantages over open surgery (Vajsbaher et al. 1 in Cogn Syst Res 64:08, 2020). If we look at the evolution from open surgery to laparoscopy to robotic surgery [2], we see a shift from relying a lot on haptic feedback in open surgery [3] to using visual feedback in robotic surgery [4] and laparoscopy providing a partial combination of both stimuli. In [5], it is argued that the importance of haptic feedback during robotic surgery is controversial. It is also hypothesized in that study that experienced surgeons are able to identify visual cues that help them to not apply excessive force to tissue during a manipulation task. On the basis of this, we form our research question for this review as: which visual cues are used by expert surgeons for manipulation tasks of soft tissue and can we use this for surgical education? After a short summary of the methodology applied in this review and the key words used to find the relevant literature as part of the Introduction, Section 2 focuses on soft-tissue manipulation in minimal-invasive surgery (MIS). Here, the visual cues found in the literature are described in detail as well as how they are used in the visual process of surgeons during surgery. In Section 3, we categorise the literature and thereby identify potential gaps in research. Here, we construct a matrix of the main literature in a content dependant order. For that, we have identified and used features of the related research that provide us with useful information to categorise the literature with regards to our research question. Current research focuses mainly on what and how we can teach robots or computer vision systems to do or see what we humans can. Some researchers however think that this is not a one-way situation, but that robots and automation systems can augment the way we learn and educate [6]. In this particular case, there is the question on how robotic systems can enhance the education of surgeons, making it more effective in terms of time, cost, and outcome. That is why, this paper also reviews a considerable amount of related literature on computer vision, neural networks, and robotic surgery applications instead of reviewing only medical research. From that, we hope that we can gain a broad understanding on how to solve surgical and educational problems with the aid of visual cues. For this review, we searched Web of Science, PubMed, and Google Scholar for the most cited papers in the area of visual cues in surgery and we used a backward snowballing approach, i.e., looking at the citations of a paper [7]. The main terms we searched for included: “visual cues in surgery”, “visual feedback in surgery OR surgical education”, and “visual cues for manipulation”.

## Methodology

**Fig. 1:**
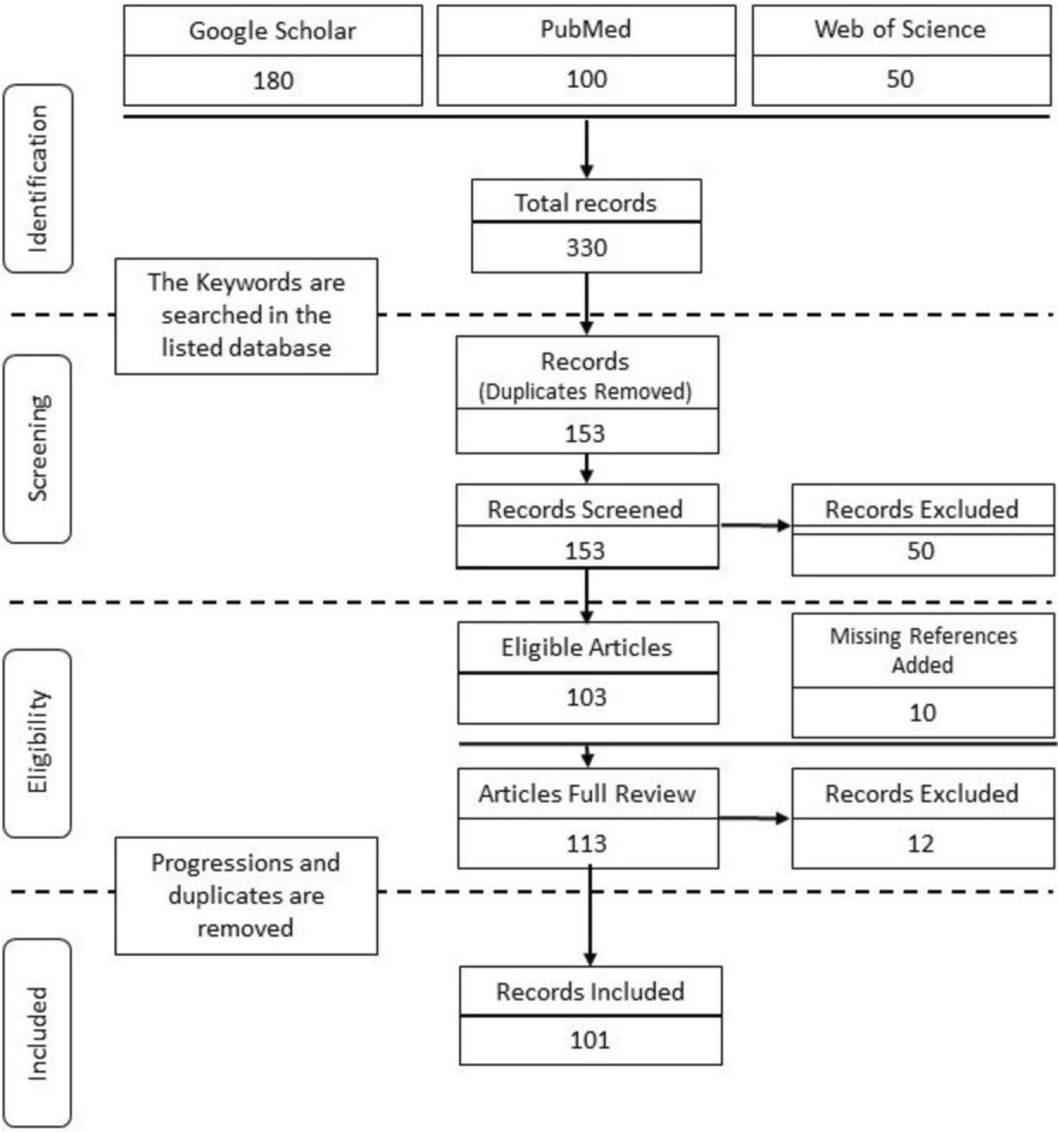
PRISMA flow diagram

The period for this review was over a year. The following keywords were used for identification: visual cues in surgery, visual feedback in robotic surgery, and visual cues in surgical education. We followed the conventions of the PRISMA flow diagram for paper selection (see Fig. 1). For screening, duplicates were removed and the remaining 153 records were skimmed through to mark the ones that are relevant to our topic. During that process, 50 records were excluded. A total of 103 articles were found eligible for a complete review. Throughout the review, the references of the selected papers were surveyed and the relevant articles that were not initially identified were added, thus increasing the total number of eligible record to 113. The work progressions and duplicate publications were removed to lead to the 101 articles included in this review. From the 101 referenced records in this review, 16 were put and sorted inside a table for an easier comparison and overview of the papers’ relevancy regarding the different visual-cue-related subtopics that we defined in this review (see Table 1).

**Table 1:** Matrix of main papers under review

		Scope	Underlying theory		Research method			Visual cue applications			Tissue manipulation	
	Paper	Specific visual cues identified	Visual feedback as surrogate for haptic feedback	Sensory substitution	Modelling of visual cues	Video analysis	Subjective two- choice experiment	Educational design solution (Training etc.)	Identification of tissue characteristics	Motion planning/navigation	With VC	Without VC
VC Identification/application in education	[25]	+			+	+		+	+			+
	[5]	+	+	+				+				+
	[40]	+				+		+	+			
VC identification	[20]	+	+	+		+			+	+		+
	[86]	+			+	+			+			+
	[63]	+	+	+					+			
	[34]	+	+	+								+
	[24]	+	+				+		+			+
	[38]	+	+	+			+		+			+
Skill/performance	[68]					+						+
	[87]					+			+			+
	[92]				+	+			+			+
	[88]					+		+				+
	[89]		+	+		+						+
	[90]				+							
	[91]			+								

## Soft-tissue manipulation in MIS and robotic surgery

**Fig. 2:**
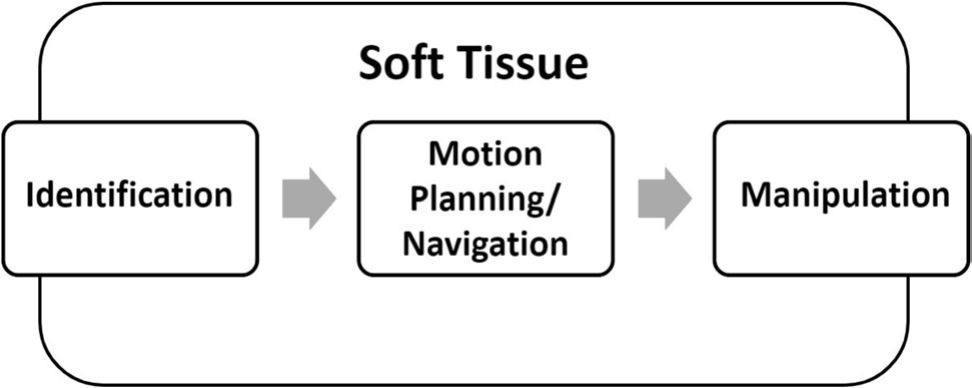
Visual process during surgery

The visual process of a soft-tissue manipulation task of a surgeon can be divided into three different mechanisms [8]: Identification [9], Motion Planning/Navigation [9–11], and Manipulation [9], (see Fig. 2). The surgeon switches constantly between these three modes during the surgery of soft tissue. The surgeon switches constantly between these three modes during the surgery of soft tissue.

Inferring information from visual feedback is essential during MIS and RS due to the distorted haptic feedback as mentioned before. It is therefore important to study the effects that surgeons have to face because of it. One phenomenon is that it gets harder for the surgeon to estimate how much force is actually and how much force needs to be applied, without the intuitive sense of touch [12]. In [13], the authors were studying the effects on gripping force with total lack of haptic feedback. They were numbing the tactile sensing of participants by anaesthetising their hands. The participants were still able to manipulate the objects successfully without slippage but with the result of an increased gripping force applied to the objects. The direction in which forces are applied to the tissue matter in terms of visual accessibility to the observer. Therefore, it can be hypothesised that everything that is done by moving the tool in the x–y-plane is easier to assess for the viewer than for example a gripping force that is in direct contact interaction with the tissue without moving it in a particular direction.

### Visual cues in surgery

The literature defines visual cues in surgery as key points or features of visual information presented to the observer, that are used to make judgements or predictions about the behaviour of tis- sue. During this literature review, we have identified several visual cues that were used either in the context of computer vision tasks or that were quantitatively analysed by surgeons or researchers in an educational context. The phrasing ‘visual cues’ is used in different contexts within the literature. There are visual cues that are given as raw data from the visual scene of the laparoscope or there are artificially induced visual cues [14] that are used to provide the surgeon with a visual representation of the forces (visual feedback) [15], e.g., pseudo-haptics or augmented-reality features. This literature review is concerned with the visual cues that occur as visual sensory input to the surgeons whilst operating. Since both laparoscopy and robotic surgery have less or no haptic feedback compared to open surgery [16], surgeons have to rely more or only on visual cues to make judgements about the applied force or tension to tissue [17]. There have been several studies that show that the lack of haptic feedback does not affect the performance of surgeons [18, 19] or lead to more tissue injury. Visual feedback can act as a surrogate for haptic feedback [20]. The following visual cues have been identified during the review of the literature to play a role during minimal-invasive surgery:

- Depth cues [21, 22]
- Colour changes [20, 23]
- Texture [20]
- Elasticity and stretchability [24]
- Reflectance [25, 26]
- Shades [25].

These visual cues can be subdivided into more specific key features of visual perception of the operative scene. It must me noted that these features cannot be viewed in isolation but can influence one another or are closely interconnected.

#### Depth cues

There is a distinction between the binocular visual cues the surgeon gets only in 3D vision [27] and the monoscopic (2D) visual cues [28]. Monoscopic visual cues are

- Motion parallax
- Relative position
- Accommodation
- Familiar size
- Object interposition
- Texture gradient
- Aerial perspective.

There are controversial results in the literature about whether 3D vision facilitates depth cue interpretation tasks in the context of laparoscopy [29]. The authors of [30] compared to 2D and 3D depth cues in surgical skill acquisition in novices. They found that additional binocular cues in stereoscopic visualisation lead to cognitive overload of the novices. In contradiction to their hypothesis, novices did not perform better with additional binocular cues. Another study, on the other hand, found that 3D visualisation significantly enhanced performance of participants given phantom surgical tasks [31]. The study in [32] claimed that their results show an improvement in skill acquisition of novices in laparoscopy due to 3D vision implementation. Participants were able to perform more complex laparoscopic tasks in a decreased amount of time and with fewer errors. One explanation for the contradicting results from different studies investigating if 3D visualization can improve surgical performance may be that the performance is very much task-dependant and experience-dependant [33]. This means that binocular cues from 3D vision might be more important in tasks that are considered to be more complex such as knot-tying, but are less important in basic tasks such as pegboard transfer. In other words, this suggests that the importance of 3D vision depth cues depends on task complexity.

Another example of a depth cue or 3D orientation cue is the “alignment of suturing material” as described in [34].

#### Colour changes

Colour patterns and changes play an important role during identification, navigation, and manipulation of soft tissue [23].

An example given by [34] for a colour-defined visual cue is “discolouration and deformation of the bowel during grasping”. The grasping instrument interrupts the blood flow in the area of the grasp and the delicate, very compliant tissue of the bowel changes colour from a darker to a lighter reddish tone. It must be noted that we found a large amount of literature about the identification of tissue characteristics with the aid of colour change cues, e.g., the identification of certain organs or the identification of injuries and tissue abnormalities. What we did not find was the application of colour change cues in tissue manipulation. The only example we found was the one given above about the bowel grasping task. To the best of our knowledge, there is no research on how exactly different tissues change colour when manipulated (e.g., the tissue gets lighter when it is pulled).

#### Texture cues

Texture can be a depth cue or a visual cue in itself [33, 35]. It is also closely correlated with reflectance and shades, since humans perceive texture through reflectance and shadow cues. Texture is a crucial visual cue to distinguish vital organs, which tend to exhibit a narrow variety of colour [25]. The shape of an instance in the operative field can be inferred from the texture. By analyzing the distortion of the texture projected in an image, the 3D coordinates of a surface in a scene are recovered [36]. The authors of [36] explain that texture distortion is measured by assuming a property of the object such as homogeneity, isotropy, or spectral content, on the original texture of an object. Then, the prior information based on the original is compared with the properties of the texture in the observed image.

#### Elasticity and stretchability

The elasticity of the tissue helps surgeons to make judgements on how to manipulate the tissue in a safe manner. With the knowledge about tissue stretchability and local tissue deformation, surgeons can estimate how much tension and force can be used in a pulling, gripping, retraction, or needle insertion [37] without risking tissue injury or tearing [5]. Another elasticity-related visual cue is the tension of suturing material used on the anatomical structures [34]. The tension of the suture material is easier to access than the surrounding soft tissue because of its contrasting colour. The literature suggests that in the event of a discrepancy between the present visual and haptic cues, humans tend to rely more on visual cues to judge the softness of compliant objects [38].

#### Reflectance and shades

It could be argued that reflectance and shades are a subcategory of colour changes, but since they play a special role with respect to navigation and manipulation, they are worthy mentioning separately.

**Fig. 3:**
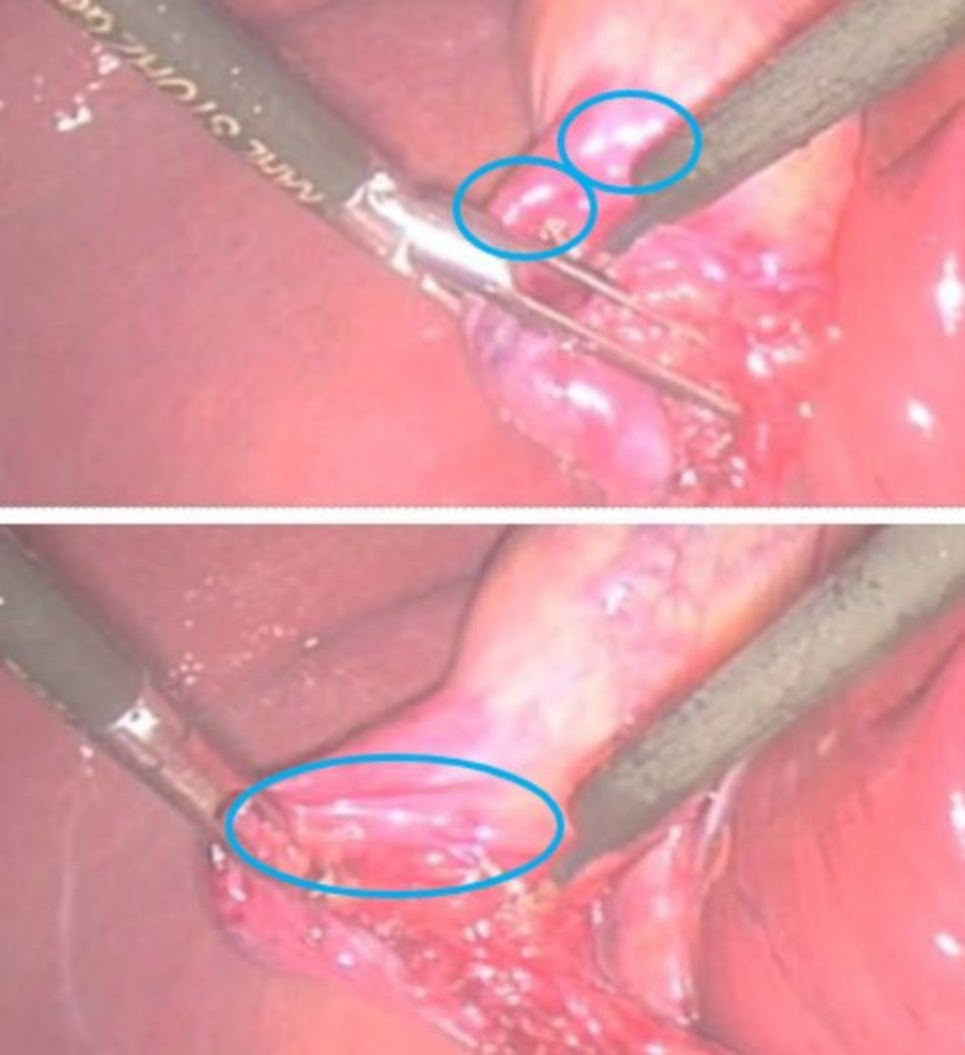
Changes in specular reflection during soft-tissue retraction (still images extracted from Video Clip S6 of the Supporting Material from [40]); (top) tissue is not stretched, (bottom) tissue is pulled

From specular highlights, surgeons can infer the texture and the elasticity of tissue as well as position of the endoscope. Cues of specular reflectance are derived from binocular disparity, motion in- formation, and the properties of highlights [26]. These properties are brightness and geometry of the highlights relative to diffuse shading on the surface [39]. When you comparing the two pictures in Fig. 3, you can see a very particular change of reflections where the tissue is pulled. The tissue in the top picture that is not pulled has more round reflections, whereas in the second picture, these reflections become a sharp white line that runs exactly along the edge of the stretched tissue. These change of reflection shape can be picked up easily by a camera system.

#### Identification and navigation

During our research, we found that visual processing by a surgeon can be divided into three different decision-making processes: *Identification* of tissue [20, 41] (e.g., classification of different organs, identification of dissection planes), *Navigation* in the operational field [42] (e.g., angle and position of the endoscope or path planning of cutting pattern), and *Manipulation* of the tissue [43] (e.g., gripping and pulling in direct contact with the tissue).

An essential precondition for the safe manipulation of tissue is the thorough and reliable identification of the correct tissue components as well as a detailed motion and navigation planning by the surgeon. In [44], it was found that most errors during MIS were related to surgeon perception, i.e., due to incorrect identification or no visual perception of the impacted structures. In the context of this literature review, it is interesting to notice that the authors of the above study proved the visual aspect of the operation to be more prone to lead to errors than motor skill imprecision of the surgeon. To identify a pathology of tissue, findings showed that visual cue interpretation during laparoscopic cholecystectomy could relate to the identification of different pathological states, such as distinguishing the appearance of chronic inflammation from that of normal tissue [40]. The authors of [40] analysed several learning situations of laparoscopy, by recording audio as well as video material of two operating surgeons, one being a senior the other being a trainee in real laparoscopy. In these instances, the trainer might use an adjective or simile to draw the trainee’s attention to subtle changes in appearance. For example, after remarking that the gallbladder wall appears adenomyotic (adenomyomyosis is a rare disease of the gallbladder characterised by epithelial proliferation and the formation of mucosal pouches through the thickened muscular layer of the gallbladder wall), he uses the adjective ‘marbled’ to draw the trainee’s attention to the visual features. It was observed that in cases that were more difficult, with more complex anatomy, more time was spent on ‘interpreting visual cues’. It must be noted that the visual descriptors used by the trainer were solely about tissue identification rather than about describing and teaching the right manipulation cues.

#### Modelling of visual cues

There are several approaches to the modelling of visual cues [45]. Many have used models of visual cues for video segmentation of laparoscopy videos [46–48]. The segmentation of the videos could then be used to improve surgical training for students. The automatic classification of different procedural steps of a recorded laparoscopy taught students how to identify each step of an operation in a cost-effective way.

(i) *Elasticity*. Mass–spring models [49] and finite-element modelling are two standard techniques used to simulate the visualisation of soft-tissue deformation during rigid-tool/soft-tissue interaction on the soft-tissue computer model. Both techniques have some disadvantages: mass–spring models ignore the impact of the indenter diameter on the soft-tissue deformation, whilst the use of finite-element modelling cannot usually achieve real-time performance due to high computational complexity [50].
  There are several virtual models of soft tissue that are used in VR simulators [51–54]. One strategy used to visualise haptic feedback is the integration of pseudo-haptics. The concept of pseudo-haptics uses visual feedback such as active cursor displacements to create the visual illusion of actual force feedback. This approach can be used to model compliance or elasticity of tissue visually. Since pseudo-haptic feedback generates virtual forces through visual feedback only, it is considered to be a cost-effective alternative to conventional haptics solutions [50]. Pseudo- haptics has mainly been investigated in the context of palpation tasks [55], e.g., identifying tumour nodes in a tissue. The authors of [50] combined Pseudo-haptics with force feedback and it was found that this combination of feedback methods performed as well as manual palpation of the tissue.
(ii) *Colour*. The paper [25] identified several visual cues in videos, which were assumed to be used by surgeons for inferring information. The authors classified the visual cues as local and global descriptors of the scene. A feature space was created with the visual cues to depict the principal axes of variability. Then, a classification model was used to segment videos of surgery.
  To detect specific steps of a surgical procedure from videos, colour features can be used in several ways. Pixel values can be used as features directly. In [25], the authors used RGB/HSV components to augment both the local descriptor (colour values) and global descriptor (colour histogram).
(iii) *Position*. Relative position of organs and instruments is an important visual cue. The paper [25] encoded the position of SURF detected keypoints with an 8 × 8 grid sampling of a Gaussian surface centred around the keypoint. The variance of the Gaussian defines the spatial “area of influence” of a keypoint.
(iv) *Shape*. Shape is an important visual cue for computer vision applications. It is used for example in detecting instruments in educational videos of surgery. The cues help to identify and thereby segment the phase of the surgery video. Shape can be encoded with various techniques, such as the Viola–Jones object detection framework, using image segmentation to isolate the instruments and match against artificial 3D models, and other methods. [25]
(v) *Texture*. Texture is a crucial visual cue to distinguish vital organs, which tend to exhibit a narrow variety of colour. Texture for example can be extracted using a co-occurrence matrix with Haralick statistical features, by a sampling of representative patches to be evaluated with a visual descriptor vector for each patch, and other methods. [25]

#### Manipulation

Manipulation is the interaction with an object in direct contact with another object that is applying a force and thereby controlling the objects movement or behaviour [56]. It can be assumed that a prediction of the behaviour of an object just from visual feedback alone is much harder to do for manipulation tasks as it is for identification or navigation tasks [57]. This is because at the contact points during manipulation, the tool that one is manipulating with is occluding the object being manipulated in the contact point. Since occlusion means one is getting no visual feedback at the point of occlusion, these points form gaps of sensory information, if one receives only visual information.

### Visual cues in surgical training

There is a manifold of different surgical training techniques such as virtual reality simulators [58], wet labs, box trainers, video-based training, or augmented-reality simulators, all of which deliver different visual cues to the observer. Virtual reality simulators are aiming to capture the reality of soft-tissue behaviour as closely as possible [59, 60]. Whilst the progress that has been made in modelling reality virtually is important in the pursue of finding low-cost solutions for surgical training, fine details of the real operating scene are still very hard to capture. Until now, the visual cues perceived during minimal-invasive surgery remain to be seen only on recorded endoscopic videos.

Cope et al. (2015) stress the importance for students to learn visual cue interpretation [40]. Mastering visual cue interpretation means having a certain amount of mental exemplars of visual cues and their contextual meaning stored in their memory (see section on sensory substitution). The more experience the surgeon has, the richer their memory about visual cue interpretation [61, 62]. When considering the practical implications of the above, the study [63] stresses that students face the problem of increasingly restricted training hours. The authors argue that this can make it more difficult for students to acquire a rich memory bank of visual exemplars [64] to be used to make judgements during MIS and RS.

**Fig. 4:**
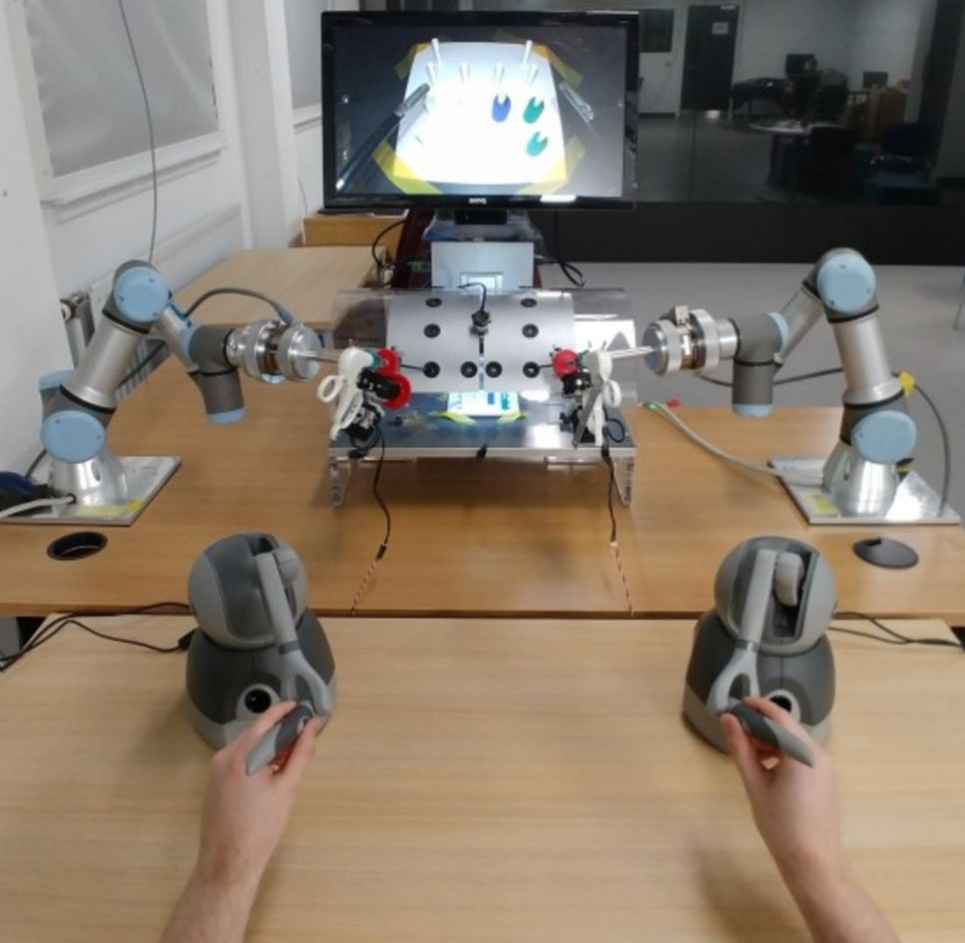
An experimental low-cost Robotic Surgery Training setup at Heriot-Watt University

Simulation-based learning methods are an important part of the skill acquisition process for MIS and RS and useful when trying to find cost-effective solutions that are available to students whenever they need training hours. However, “the identification of the plane for dissection—made by interpreting subtle differences in colour or texture of the tissues, and how they dynamically re-spond to tension—can seldom be adequately simulated” [63]. Even though current simulation techniques have made immense progress with respect to accuracy, they are still an estimation of reality [65] using different modelling techniques. Therefore, the study concludes that “one potential avenue for surgical education is the design of educational interventions that specifically address visual cue interpretation” [65]. There are several educational design solutions that aim to make medical education more cost-effective and available to a broader audience. Fig. 4 depicts a robotic training system from Heriot-Watt University that is designed as a low-cost training solution for robotic surgery. A substantial amount of simulators like the ProMIS (Haptica) or LapMENTOR (Simbionix) were introduced during the last two decades to cover every aspect of surgical training. Considering the conventional measures of surgeon performance used in training platforms, such as time taken, instrument path length, and smoothness of motion might not be the best choice to measure surgical ability in terms of result-oriented skill assessment [66]. The authors of [63] suggest that “appropriateness of the surgeon’s actions” might provide a more sensible metric for the measurement of the quality of surgical ability. The simulators for example evolved to analyse if the surgeon cuts in the correct place rather than only measuring completion time or other quantitative metrics [67]. When these errors are also considered in the context of their severity, the paper [67] argues that one can reliably differentiate between expert and novice surgeons. This brings us to the conclusion that qualitative metrics can have more impact in surgical training than quantitative metrics like completion time or smoothness of movements. This could be an indication, that if we can identify and define visual cues that are actually used by expert surgeons to manipulate tissue safely, we could potentially enhance surgical training in a meaningful way. The appropriateness of certain actions could potentially be defined more clearly by building a library of visual cues that is found to be sensible by expert surgeons and maps manipulation actions of soft tissue to the appropriate visual cues.

### Error mechanisms and performance measurements

In [68], the authors studied what kind of errors happened and how frequently they occurred during laparoscopy procedures, what manipulation mechanism had led to the error, and what consequences were incurred. The authors analysed 50 videos of laparoscopies from different surgeons. The most frequently observed errors were: use of too much force, too much distance between tool and tissue, "inadequate visualisation and wrong orientation of the instrument or dissection plane. These errors led to different injuries with varying severity. The error mechanisms can be measured visually and thereby it can be concluded that the right set of visual cues should be studied, matched to the respective situation, and used for educational purposes to improve student’s learning outcome or to aid the performance of fully trained surgeons. Another factor that could play a role in the learning process of MIS is the oblique effect [69]. It can be hypothesised that expert surgeons learn to compensate for the errors that the oblique effect produces. Human perception is more accurate for vertical and horizontal movements than for oblique ones. This effect leads to a misperception of the direction of motions. This, in our opinion, stresses the importance of a stable movement and fixation of the endoscope with a stable horizon whilst learning to manipulate tissue with tools. This will help students to learn how to use velocity redundancies that can be used to compensate for the oblique effect.

It is not an easy task to estimate the interaction forces and reactions in an ongoing contact task visually [70, 71]. When studying how visual cues can be exploited one keep in mind that sensory input for a surgeon from different sources are interconnected and the surgeon will have learned to interpret, perhaps unconsciously, this complex set of feedback [72], i.e., one sensory feedback influencing the other in the context of motor control [73]. An interesting study was conducted by Adams et al. (2013), where the subjects were only given visual feedback whilst handling an object in their hands. When studying the effects of interrupting haptic and tactile sensory information of subjects, the study [13] found that the subject’s gripping force increased significantly. They injected local anaesthetics into the hands of the subjects and made them grip objects with only visual feedback helping them control their actions. They concluded that the increased gripping force is a strategic response of the nervous system to secure a grip without slippage of the object despite the deficit of sensory information. On the other hand, another study [74] that was researching ways to estimate forces applied to soft tissue with vision-based methods found that in a wet lab experiment, it was possible to predict how much force was applied to the tissue with a mean absolute error of 0.814 N. However, the model was trained on the indentation force of the tool only. Indentation force means an object is indented with a one-directional force, whereas a gripping force has two counteracting forces. It seems logical that an estimation of gripping force from vision only is inherently harder [75], since one side of the gripping tool tip is occluded by the tissue or the visually perceptible changes are much smaller, since the applied force is interacting from two directions making it harder to visually estimate elasticity. The model described above was exploiting the elasticity of the tissue as a crucial visual cue. However, if the tissue is gripped and pinned inside the two counteracting sides of the tool tip, there is less deformation to be observed on the tissue, hence again making gripping force prediction more difficult to predict visually than one-directional indentation force [76]. Several studies have tried to implement objective measures for the skill differentiation of expert surgeons and trainee surgeons . The metrics in the literature include: time to complete a particular defined surgical task [77, 78], hand path length [78, 79], number of movements [80], smoothness of hand movements [81, 82], and force–torque signatures of particular movements [63]. Another metric is eye tracking. The studies [83] and [84] found that there is a reliable distinction in eye movement patterns between expert and novice surgeons. A critical review of the metrics above has been given in Section 4, “Visual Cues in Surgical Training”.

### Sensory substitution

Hagen et al. (2008) investigated sensory substitution of haptic feedback with visual feedback [34]. They concluded that real haptic feedback is not a necessity for expert surgeons to perform MIS or RS in a safe manner . The more experience a surgeon has, the more they experience something that the authors of [20] called the ‘reverse Braille effect’. Therefore, these papers argue that visual cues can act as a surrogate for haptic feedback [5, 20, 34]. The authors of Hagen et al. (2008) also investigated the effect of the absence of haptic feedback in robotic surgery and found that the surgeon at the console “learns” to translate optics into tactiles subconsciously and is able to use this information in the course of the procedure’ [34]. They described this phenomenon as “a neurological form of conditioning”. This conditioning provides the surgeons with a mental heuristic that estimates how much force is applied, because the surgeon connects the visual cues of the applied force with a certain “feeling”. An experienced surgeon would have a richer understanding [61, 85] (consciously or unconsciously) of how a specific grip on a piece of bowel would lead to a specific amount of discolouration, how that discolouration would “feel”, and therefore how much force they apply. From a neurological perspective, the concept of sensory substitution can be explained by how humans perceive. Human perception can be described as a weighted sum of sensory input. According to the study in [63], this means that surgeons combine information from visual and haptic cues, weighting them depending on the context and the quality of the cues available to them. If the quality of visual feedback perceived by an observer is higher than the quality of haptic feedback perceived, than it would follow from the above that the decision is determined mainly by the visual stimuli. There are several studies that support the assumption that haptic feedback learned first in detail, by touching and manipulating the tissue in open surgery or wet labs and then being less present in laparoscopy, can be translated mentally into visual cue interpretation sufficiently.

## Review of visual cue identification and applications

In Table 1, we have collected the most relevant studies related to the concept of visual cues in surgery and present it in the form of a matrix that relates the studies to a number of factors we have identified. The rows of the table correspond to the studies identified as most relevant to this review.

The rows, hence the collection of studies, have been grouped under three categories: Skill/Performance, VC identification, and VC Identification and application in education. The first category is composed of three papers that include an educational design solution in which they apply the visual cues they identified in their study [5, 25, 40]. The second category shows research papers that are mainly concerned with the identification of visual cues but have no educational application with respect to visual cues [20, 24, 34, 38, 63, 86]. The third category summarises the work that has focussed on skill or performance of surgeons without the context of specific visual cues but with visual components such as video analysis. Since we want to investigate the connection between visual cues and their application in surgical education, it seemed sensible to review the literature on how surgeon’s skill and performance is actually measured. Therefore, the third category is a collection of papers that investigate error mechanisms of surgeons as well as good performance measures (see also Section 3.3 [68, 87–91]).

The columns of Table 1 correspond to the factors we use to examine and compare the literature. The first column of the table indicates the research that has identified specific visual cues in their work, for example analysing specific colour patterns or identifying depth cues. The second column “Visual Cues as a Surrogate for Haptic Feedback” depicts the papers that argue that expert surgeons rely heavily on visual feedback when compared to haptic feedback. They assume that the need for haptic feedback is overestimated by novices and that it can be substituted by visual cues in an efficient way. The column “Sensory Substitution” is closely related to the previous column, but it is more general. Whilst the previous column focuses on papers with experimental application of haptic feedback substitution, the category in the third column refers to the papers that have some aspect of comparison between the use of haptic feedback and visual feedback during surgery as well as being related to the theory behind sensory substitution (see section 3.4). The next three columns are summarised under the category “Research methods”. They mark which papers have used which research methods for their studies. The three research methods that were identified during this review are “Modelling of visual cues”, “Video analysis”, and “Subjective two-choice experiment”. It is noticeable that video analysis is the main method used, especially in surgeon skill and performance research. Furthermore, it can be noticed that most papers, even if they identified specific visual cues, did not proceed to modelling the visual cues for further application. The next category, “Visual Cue application”, is composed of three columns. Here, we mark which of the reviewed papers have an educational design solution, which identified characteristics of tissue, and which include motion planning or navigation with the aid of visual cues.

As the reader can see from the table, most of the papers remained in the first stage of identification of visual cues and visual characteristics of tissue. Some of them used these results to create an educational design solution, and only one of them used the visual cues for motion planning or navigation tasks. Although it is part of the visual process of surgery (as you can see from Fig. 1), we chose to make the last category “Tissue Manipulation” a separate category rather than including it in “Visual Cue Applications”. This is because we wanted to show that, although there are a lot of papers about tissue manipulation, none of these study the impact of visual cues on tissue manipulation. This depicts the lack of research in vision-focussed tissue manipulation.

## Comparative recap of the review

The knowledge we gain from the matrix of Table 1 and from the review of the literature in general point to a lack of research in the domain of visual cue application for manipulation and education. The question we ultimately ask is: what does the review tell us for potential future research? To answer this question, we clustered the papers contentwise and categorised them in a way that exemplified the lack of research in visual cue application for soft-tissue manipulation and that showed an emphasis on visual identification of tissue in the current literature. Since a large amount of the literature is also about the application of haptic feedback [93] [94] in MIS or surgical training, it is sensible to comment on the relationship between haptic and visual feedback and how they can be connected for educational purposes. Ström et al. (2006) have shown that early exposure to haptic feedback enhances performance in surgical simulator training significantly [95]. It can be hypothesized that a withdrawal process [96] of haptic feedback towards the use of visual feedback only could be beneficial for the learning process.

## Conclusion

This literature review aims to add value by summarising empirical insights on visual cues in surgery and thereby providing a synthesis of what is already known and what is not. The main output is to reflect the state of knowledge in current research and show potential gaps in the literature [7]. The literature reviewed in this work investigates visual cues and their application in surgical education. The clustering of the papers showed explicitly that state-of-the-art research does not in particular study the direct effects of visual cues in relation to manipulation of the tissue and training for that purpose but is more concentrated on tissue identification. Therefore, there seems to be a gap in literature about the use of visual cues for educational design solutions, that aid the training of manipulation of soft tissue in MIS and in RIS [97]. By addressing that gap, visual cue identification could be made more accessible and set it in the context of manipulation tasks. Approaches such as e-learning environments for medical education are limited in providing the realism needed to train students [98]. These environments are particularly limited to provide the students with the necessary feedback of visual cues whilst performing the task. To address these problems, advanced visual cues could be used to improve medical training and learning performance as well as potentially produce better surgery outcomes. More accurate visual cues can enable better decision making by trainees and surgeons alike whilst performing soft-tissue manipulation tasks. A good knowledge of visual cues and skills to interpret those in a correct and useful way might result in less damage to the manipulated tissue and give trainees and surgeons more confidence in their ability to manipulate soft tissue safely.

## Data Availability

No datasets were generated or analysed during the current study.
